# Fructose‐Induced Impairment of Liver and Skeletal Muscle Metabolism Is Prevented by Administration of *Shouchella clausii* Spores by Preserving Mitochondrial Function and Insulin Sensitivity

**DOI:** 10.1002/mnfr.70063

**Published:** 2025-04-10

**Authors:** Angela Di Porzio, Valentina Barrella, Anella Saggese, Loredana Baccigalupi, Luisa Cigliano, Ezio Ricca, Susanna Iossa, Arianna Mazzoli

**Affiliations:** ^1^ Department of Biology University of Naples Federico II Naples Italy; ^2^ NBFC National Biodiversity Future Center Palermo Italy; ^3^ Task Force on Microbiome Studies University of Naples Federico II Napoli Italy; ^4^ Department of Molecular Medicine and Medical Biotechnology University of Naples Federico II Naples Italy

**Keywords:** fructose, inflammation, mitochondria, oxidative stress, probiotics

## Abstract

The objective of the study was to evaluate the efficacy of *S. clausii* spores (SF174) in counteracting the deleterious effects of dietary fructose. Thirty‐days old male Wistar rats were treated for 6 weeks: control group: 0.5 mL of 10% sucrose solution (without probiotics); fructose group: 0.5 mL of 10% sucrose solution + high‐fructose diet (without probiotics); SF174 group: 0.5 mL of 10% sucrose solution containing SF174 (5 × 10⁹ CFU) + high‐fructose diet. Fructose intake induced an increase in proinflammatory cytokines in portal plasma, liver, and skeletal muscle, a decrease in insulin sensitivity in both tissues and a condition of hepatic steatosis. An increase in the mitochondrial activity in the liver and a decrease in skeletal muscle were evidenced, together with an increase in the thiobarbituric acid reactive substances (TBARS) levels and a decrease in the antioxidant enzyme activity. All the above alterations were counteracted by probiotic administration. We here demonstrate for the first time that *S. clausii* SF174 counteracts low‐grade inflammation and insulin resistance induced by fructose, protects mitochondria from changes in oxidative capacity, and maintains unaltered the oxidative balance. Therefore, *S. clausii* SF174 administration can be an effective strategy to prevent the unhealthy consequences of dietary fructose.

## Graphical abstract

*S. clausii* SF174 spores were effective in preventing the onset of low‐grade inflammation and insulin resistance induced by fructose‐rich diet in young rats, preserving the mitochondria from changes in the respiratory capacity in both liver and skeletal muscle, maintaining unaltered the oxidative balance, thus counteracting the deleterious effects of dietary fructose.

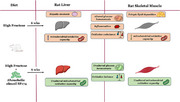

**Abbreviations**
ALT: alanine aminotransferase
DSM: Difco Sporulation medium
FAS: fatty acid synthase
HFCS: high‐fructose corn syrup
HIRI: hepatic insulin resistance index
IL‐6: interleukin‐6
IL‐10: interleukin‐10
MISI: muscle insulin sensitivity index
ROS: reactive oxygen species
SF174: *S. clausii* spores
SOD: superoxide dismutase
TAG: triglyceride
TBARS: thiobarbituric acid reactive substances
TNF‐α: tumor necrosis factor‐alpha

## 1 Introduction

Unhealthy diets are major drivers of noncommunicable diseases, becoming the major health hazard of modern world [1]. Fructose consumption has increased due to the intake of sucrose or high‐fructose corn syrup (HFCS), which is present in foods and beverages such as soft drinks, sweets, bakery goods, and dairy products. Recent studies reported that the increased intake of fructose could contribute to the development of metabolic abnormalities, even in the absence of weight gain [2].

Increased fructose intake has been associated with alteration of the gut barrier permeability [3], leaky gut, and subsequent endotoxemia [4]. The resulting inflammation can strongly impact metabolically relevant organs, namely the liver and the skeletal muscle [5]. Indeed, even a short‐term fructose diet has been shown to alter both liver and skeletal muscle metabolism, inducing insulin resistance, oxidative stress, and alteration of mitochondrial oxidative capacity [6, 7].

The use of probiotics as a therapeutic approach has grown exponentially over the last years [8]. In particular, strains of *Shouchella clausii*, previously indicated as *Bacillus clausii*, have been used in a range of studies that highlight their useful physiological properties, such as heat‐, acid‐, and bile salt‐tolerance, vitamin synthesis, and a broad‐spectrum antibiotic resistance that cannot be genetically transferred to other species [9]. The key characteristics of this species include the ability to form spores, with a following tolerance to heat, acid, and salt ensuring a safe passage through the human gastrointestinal tract [10, 11]. *S. clausii* spores (SF174) have been shown effective in treating persistent diarrhea in children [12], irritable bowel syndrome [13], and in favoring gastrointestinal health [14], with an immunomodulatory and antioxidant effect [15]. However, no studies evaluated the efficacy of SF174 on diet‐induced metabolic diseases.

We have recently shown that the administration of SF174, a specific strain of *S. clausii*, was effective in preserving gut functions in young rats that received 6 weeks of high fructose diet, mainly by avoiding the onset of gut inflammation, with only a minor effect on the reshaping of gut microbiota [16]. We here decided to extend our study by evaluating the efficacy of *S. clausii* SF174 in counteracting the deleterious effect induced in the liver and the skeletal muscle by a high fructose diet, since previous studies evidenced the link between inflammation, gut microbes, and internal organ dysfunction [17, 18, 19, 20, 21]. In particular, we focused on insulin sensitivity, inflammation, oxidative stress, and mitochondrial oxidative capacity in the liver and skeletal muscle.

## 2 Materials and Methods

### 2.1 Bacterial Strain and Spore Preparation and Purification

Cells of *S. clausii* SF174 were induced to sporulate at 37°C in Difco Sporulation medium (DSM) [22] with vigorous shaking. Spores were harvested by centrifugation (10 min; 10 000 × *g*), washed three times with distilled water, and purified as described before [23]. Cleaned spores were stored at −20°C in water, and spore counts were determined by serial dilution and plating counting.

### 2.2 Animals and Treatments

All the animal experiments were authorized by Italian Health Ministry (137/2022‐PR) and approved by “Comitato Etico‐Scientifico per la Sperimentazione Animale” of the University of Naples “Federico II”. The procedures used in this work observe the animal ethics principles and regulations of the Italian Health Ministry. The authors ensured that all steps were taken to minimize the pain and suffering of the animals.

Male Wistar rats (Charles River, Calco, Lecco, Italy) aged 30 days were caged in a temperature‐controlled room (23 ± 1°C) with a 12 h light/dark cycle (06:30–18:30 h). After 1 week of acclimation period, dietary and probiotic treatment was carried out for 6 weeks, and the composition of the two diets is shown in Table S1. Rats were randomly assigned to three experimental groups, each composed of eight rats:

- Control group (C): control diet plus 0.5 mL of 10% sucrose solution (without probiotics).
- Fructose group (F): fructose‐rich diet plus 0.5 mL of 10% sucrose solution (without probiotics).
- SF174 group: fructose‐rich diet plus 0.5 mL of 10% sucrose solution containing SF174 (5 × 10⁹ CFU).

Sucrose solution with or without probiotics was presented by an operator every day at the same hour through a needless syringe and voluntarily consumed by rats, as described in [16]. The amount of sucrose administered daily is negligible in terms of amount and energy content, since the daily amount of sucrose used for probiotic administration corresponds to 50 mg and 0.84 kJ. This amount represents 0.25% of the daily energy intake of rats and 0.42% of the daily intake of carbohydrates. Body weight and food intake were monitored during the experimental period and are reported in Figure S1.

At the end of the experimental period, the rats were anesthetized with sodium pentothal (40 mg/kg intraperitoneal, i.p.) and euthanized by decapitation. Liver and hindleg skeletal muscles (gastrocnemius, tibialis, and quadriceps from both legs) were rapidly dissected, freed of excess fat and connective tissue were used to prepare paraffin‐embedded sections or snap frozen and stored at −80°C for further analyses, as described in section 2.3,2.5 and 2.6.

### 2.3 Inflammatory Profile

At the time of sacrifice, portal plasma samples were collected in tubes containing EDTA, centrifuged at 1400 × *g* for 15 min, and subsequently stored at −20°C.

Concentration of tumor necrosis factor‐alpha (TNF‐α), interleukin‐6 (IL‐6), and interleukin‐10 (IL‐10) in portal plasma and proteins extracts from liver and skeletal muscle were assessed with dedicated enzyme linked immunosorbent assays (R&D Systems, Minneapolis, MN), specific for rats, according to manufacturer's instructions.

### 2.4 Glucose Homeostasis and Insulin Sensitivity

The glucose tolerance test was performed the day before the euthanasia. Rats were fasted (from 8 a.m.) and after 6 h the basal postabsorptive blood samples were collected from the tail vein in EDTA‐coated tubes for plasma separation. Then, glucose (2 g/kg) was injected intraperitoneally, and blood samples were taken after 20, 40, 60, 90, and 120 min.

Blood samples were centrifuged at 1400 × *g* for 8 min at 4°C, plasma was collected and stored at −20°C. Plasma glucose concentration was measured by a colorimetric enzymatic method (GS Diagnostics SRL, Guidonia Montecelio, Rome, Italy). Plasma insulin concentration was measured using an ELISA kit (DiaMetra, Boldon, UK). Hepatic insulin resistance index (HIRI) and muscle insulin sensitivity index (MISI) were calculated according to Abdul‐Ghani et al. [24].

### 2.5 Liver and Skeletal Muscle Composition

Colorimetric enzymatic methods were used to assess systemic plasma triglycerides (TAGs) and alanine aminotransferase (ALT) using commercial kits (GS Diagnostics SRL, Guidonia Montecelio, Rome, Italy).

Liver and skeletal muscle TAGs were assessed using commercial kits (GS Diagnostics SRL). Lipid content was measured by the Folch extraction method, ceramide content was evaluated by ELISA, and the activity of fatty acid synthase (FAS) was measured in liver homogenates prepared in KCl 175 mM, Tris 10 mM, pH 7.5 (1:8 w/v), as previously described [25].

Liver tissue samples, after fixation in 4% buffered formaldehyde, were embedded in paraffin, cut into 4 µm thick sections, and stained with hematoxylin and eosin. To evaluate hepatic steatosis, images were acquired with 10x magnification, and 3 random field/section per rat were analyzed and scored blindly.

Immunohistochemistry was performed on dewaxed 4 µm serial sections using 3% hydrogen peroxide to inactivate endogenous peroxidases followed by normal horse serum to reduce nonspecific staining. Consecutive serial sections were incubated for 1 h at room temperature with antibodies for F4/80 (AbboMax, San Jose, CA, USA) and then incubated for 30 min at room temperature with biotinylated, goat anti‐rat IgG (Invitrogen, A) secondary antibody. Histochemical reactions were performed using the Vectastain ABC Kit (Vector Laboratories, Mowry AveNewark, CA, USA) and 3,3‐diaminobenzidine as substrate (Sigma, St. Louis, MO, USA). Sections were counterstained with hematoxylin. Images were acquired at a 40x magnification.

### 2.6 Liver and Skeletal Muscle Mitochondrial Physiology and Oxidative Stress

Liver and skeletal muscle samples were homogenized (1:1000, w/v) in Mir05 medium containing 110 mM sucrose, 60 mM K‐lactobionate, 20 mM Hepes, 20 mM taurine, 10 mM KH_2_PO_4_, 6 mM MgCl_2_, 0.5 mM EGTA, 0.1% fatty acid‐free BSA, pH 7.0. Mitochondrial function was evaluated at 37°C by using O2k (Oroboros Instruments, Innsbruck, Austria) as previously described [6, 7].

Complex II activity was assessed in homogenates from liver and skeletal muscle made in 50 mM phosphate buffer, pH 7.0 (1:50 w/v), in agreement with Spinazzi et al. [26].

Oxidative stress markers were evaluated in liver and skeletal muscle homogenates prepared in 50 mM phosphate buffer, pH 7.0 (1:50 w/v).

Lipid peroxidation was determined by measuring thiobarbituric acid reactive substances (TBARS), using the thiobarbituric acid assay as previously described [27].

Superoxide dismutase (SOD) activity was evaluated by following the decrease in the reduction rate of cytochrome *c* by superoxide radicals in a medium containing 50 mM KH_2_PO_4_ pH 7.8, 20 mM cytochrome *c*, 0.1 mM xanthine, and 0.01 units of xanthine oxidase. The measurement has been performed in absence or in presence of KCN 2 mM, able to inhibit the cytosolic SOD (SOD1) but the mitochondrial one (SOD2), to evaluate both SOD1 and SOD2 activities, in agreement with Flohé and Otting [28].

Catalase activity was measured in 50 mM phosphate buffer, pH 7.0 containing 10 mM H_2_O_2_, and 0.25% w/v Triton X‐100 by monitoring the decomposition of H_2_O_2_ at 240 nm as described previously [29].

### 2.7 Statistical Analysis

Data were reported as mean values ± SEM. The Graph Pad Prism 10 program (GraphPad Software, San Diego, CA, USA) was used to confirm that raw data have normal distribution and to perform one‐way ANOVA followed by the Tukey post‐hoc test. A probability of <5% (*p* < 0.05) was considered statistically significant in all analyses.

## 3 Results

### 3.1 Inflammatory Profile

**FIGURE 1:**
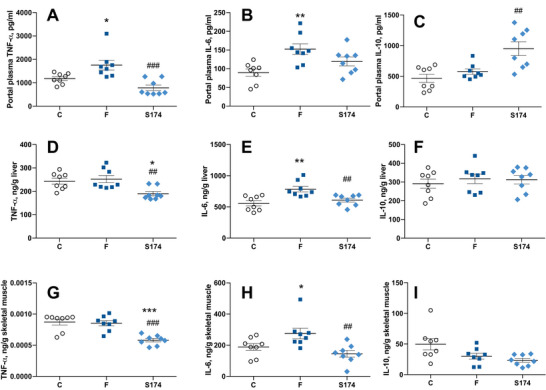
Inflammatory profile in the portal plasma, liver, and skeletal muscle. Tumor necrosis factor (TNF)‐α (A, D, G), interleukin‐6 (IL‐6) (B, E, H), interleukin‐10 (IL‐10) (C, F, I) levels in the portal plasma, liver, and skeletal muscle from rats fed control diet (C), fructose‐rich diet (F), and fructose‐rich diet plus spores of *S. clausii* SF174 (SF174) for 6 weeks. Values are the means ± SEM of eight different rats. *p < 0.05, **p < 0.01, ***p < 0.001 compared to C rats; ##*p* < 0.01, ### *p* < 0.001, compared to F rats (one‐way ANOVA followed by Tuckey post‐test).

Six weeks of fructose‐rich diet increased the levels of the proinflammatory cytokines, TNF‐α, and IL‐6 in portal plasma (Figure 1A, B), while the levels of the antiinflammatory cytokine IL‐10 were not affected (Figure 1C). In portal plasma of rats treated with SF174, TNF‐α and IL‐6 levels were similar to those of control rats (Figure 1A, B). In addition, a significant increase of the antiinflammatory cytokine IL‐10 compared to both control (C) and fructose‐fed (F) rats was observed, suggesting that the SF174 treatment had an antiinflammatory action independently from the diet regimen (Figure 1C). Portal inflammation affected the inflammatory status in the liver (Figure 1D–F) and skeletal muscle (Figure 1G–I). A significant increase in IL‐6 levels in F rats compared to C (Figure 1E–H) was observed while the TNF‐α levels were not affected in the liver or in the skeletal muscle (Figure 1D, G, respectively). The SF174 treatment showed an antiinflammatory action in both tissues with a reduction of IL‐6 levels compared to F rats (Figure 1E, H), together with a diet‐independent reduction of TNF‐α levels compared to both C and F rats (Figure 1D, G). As for IL‐10 levels, no variation was found in liver and skeletal muscle between the three groups of rats (Figure 1F–I).

### 3.2 Glucose Homeostasis and Insulin Sensitivity

**FIGURE 2:**
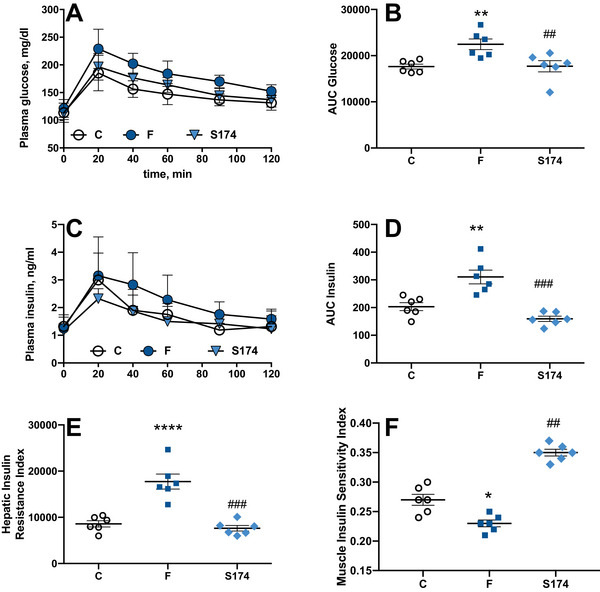
Glucose homeostasis and insulin sensitivity markers. Plasma glucose (A), glucose area under the curve (AUC) (B), plasma insulin (C), insulin AUC (D), during 120 min after intraperitoneal injection of glucose, hepatic insulin resistance index (HIRI) (E) and muscle insulin sensitivity index (MISI) (F) from rats fed control diet (C), fructose‐rich diet (F), and fructose‐rich diet plus spores of *S. clausii* SF174 (SF174) for 6 weeks. Values are the means ± SEM of six different rats. *p < 0.05, **p < 0.01, ****p < 0.0001 compared to C rats; ##*p* < 0.01, ###*p* < 0.001 compared to F rats (one‐way ANOVA followed by Tuckey post‐test).

Impairment in glucose homeostasis and insulin sensitivity was elicited by fructose‐rich diet after 6 weeks of treatment (Figure 2), while the SF174 treatment was able to counteract the deleterious effect of fructose, keeping both glucose and insulin at levels comparable to C rats (Figure 2A–D). Moreover, in F rats, HIRI was increased (Figure 2E) and muscle insulin sensitivity index was decreased (Figure 2F) compared to C rats, but these alterations were not found in SF174‐treated rats, thus indicating the ability of spores of *S. clausii* SF174 in preventing the fructose‐induced alteration of insulin sensitivity and preserving the glucose homeostasis at whole body level.

### 3.3 Liver and Skeletal Muscle Composition

**FIGURE 3:**
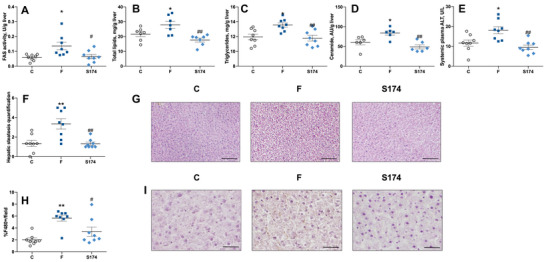
Liver composition. FAS activity (A), total lipids (B), triglycerides (C), ceramide (D), plasma alanine aminotransferase (ALT) (E), degree of steatosis (F) with representative images (G, scale bar = 100 µm), F480 immunohistochemistry quantification (H) with representative images (I, scale bar = 25 µm) in rats fed control diet (C), fructose‐rich diet (F), and fructose‐rich diet plus spores of *S. clausii* SF174 (SF174) for 6 weeks. Values are the means ± SEM of six/eight different rats. *p < 0.05, **p < 0.01 compared to C rats; #*p* < 0.05, ##*p* < 0.01, compared to F rats (one‐way ANOVA followed by Tuckey post‐test).

**FIGURE 4:**
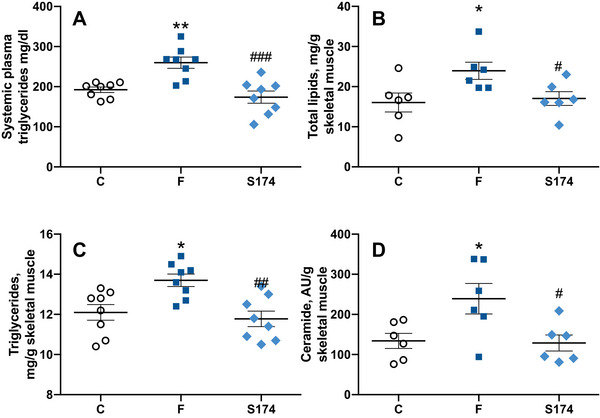
Skeletal muscle composition. Triglycerides levels in the systemic plasma (A), skeletal muscle total lipids (B), triglycerides (C), and ceramide (D), in rats fed control diet (C), fructose‐rich diet (F), and fructose‐rich diet plus spores of *S. clausii* SF174 (SF174) for 6 weeks. Values are the means ± SEM of six/eight different rats. *p < 0.05, **p < 0.01 compared to C rats; #*p* < 0.05, ##*p* < 0.01, ###*p* < 0.001 compared to F rats (one‐way ANOVA followed by Tuckey post‐test).

The increased fructose intake is able to induce de novo lipogenesis at the level of the liver [30]. In agreement, we found that FAS activity (Figure 3A), hepatic lipids (Figure 3B), TAGs (Figure 3C), ceramide (Figure 3D), and degree of steatosis (Figure 3F, G) were all significantly upregulated by fructose, with a following increase in liver necrosis, as indicated by an increase in plasma ALT levels (Figure 3E). Moreover, the increased number of F4/80 macrophages in F rats confirmed the presence of hepatic inflammation (Figure 3H, I). When the liver exceeds its capacity to deposit TAGs produced by de novo lipogenesis, they are released into the systemic circulation and eventually reach the skeletal muscle. Indeed, we show higher TAG levels in the systemic plasma (Figure 4A), together with total lipids, TAGs, and ceramide (Figure 4B–D) in the skeletal muscle of F rats compared to C rats.

The SF174 treatment prevented the hepatic de novo lipogenesis activation, the ectopic lipid deposition, the ceramide accumulation in both liver and skeletal muscle (Figures 3A–D and 4), and the onset of hepatic steatosis, necrosis, and macrophages infiltration in the liver (Figure 3E–I).

### 3.4 Liver and Skeletal Muscle Mitochondrial Physiology and Oxidative Stress

**FIGURE 5:**
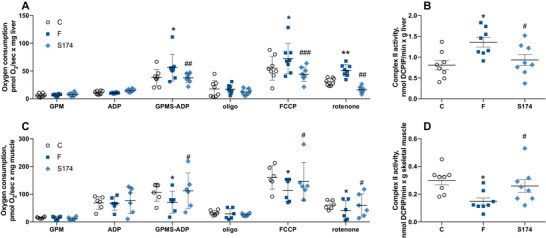
Mitochondrial physiology in liver and skeletal muscle. Respiratory rates (A, C) and Complex II activity (B, D), in liver and skeletal muscle from rats fed control diet (C), fructose‐rich diet (F), and fructose‐rich diet plus spores of *S. clausii* SF174 (SF174) for 6 weeks. Values are the means ± SEM of six/eight different rats. *p < 0.05, **p < 0.01 compared to C rats; #*p* < 0.05, ##*p* < 0.01, ###*p* < 0.001 compared to F rats (one‐way ANOVA followed by Tuckey post‐test).

Considering that mitochondria are the primary cellular site responsible for substrate oxidation [31], we studied hepatic and muscular mitochondrial physiology. In the liver, we found increased respiration driven by Complexes I and II in F rats, in the presence of ATP, while respiration driven solely by Complex I was not affected by fructose intake. In addition, when the maximal capacity of the respiratory chain was measured in the presence of the uncoupler FCCP, a significant increase was evident, even after the addition of rotenone, an inhibitor of Complex I (Figure 5A). The above increase was due to a higher activity of Complex II (Figure 5B).

In the skeletal muscle, the high fructose diet induced a decrease in mitochondrial respiratory activity, in the presence of FAD‐linked substates, ATP, FCCP, and rotenone (Figure 5C), due to the decreased activity of Complex II (Figure 5D). All the above mitochondrial modifications were not found in SF174 rats.

**FIGURE 6:**
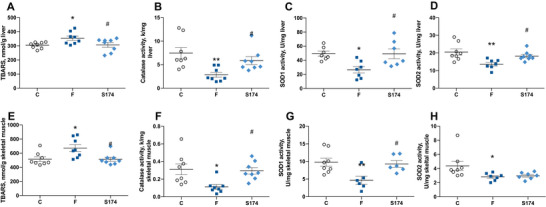
Oxidative balance in the liver and skeletal muscle. Lipid peroxidation (A, E), catalase activity (B, F), cytosolic superoxide dismutase (SOD1) (C, G), and mitochondrial superoxide dismutase activities (SOD2) (D, H) in liver and skeletal muscle from rats fed control diet (C), fructose‐rich diet (F), and fructose‐rich diet plus spores of *S. clausii* SF174 (SF174) for 6 weeks. Values are the means ± SEM of six/eight different rats. *p < 0.05, **p < 0.01 compared to C rats; #*p* < 0.05 compared to F rats (one‐way ANOVA followed by Tuckey post‐test).

Mitochondrial impairment is strictly linked to the onset of oxidative stress, since mitochondria are the main cellular site involved in reactive oxygen species (ROS) production. Indeed, fructose‐rich diet induced oxidative unbalance in both liver and skeletal muscle, as shown by higher levels of lipid peroxidation (Figure 6A, E) and the lower activity of antioxidant enzymes catalase (Figure 6B, F), SOD1 (Figure 6C, G), and SOD2 (Figure 6D, H). All the above alterations, with the exception of SOD2 reduction in skeletal muscle, were not detected in the liver and skeletal muscle of SF174‐treated rats (Figure 6).

## 4 Discussion

The main result of this study is that the administration of *S. clausii* SF174 spores protects the liver and the skeletal muscle from the development of fructose‐induced metabolic alterations in young rats and is also able to exert an antiinflammatory effect independently from the dietary regimen.

We recently found that a high fructose diet induces a disruption of the intestinal barrier, leading to increased intestinal permeability, and passage of LPS in the portal plasma [16]. Accordingly, we here found increased proinflammatory cytokines levels, namely TNF‐α and IL‐6, in the portal plasma of F rats. The condition of fructose‐induced plasma inflammation has an impact also at the level of the liver and the skeletal muscle. Interestingly, the administration of *S. clausii* SF174 prevented the fructose‐driven inflammatory response both at the systemic and tissue level. Indeed, gram‐positive bacteria, such as *S. clausii*, are able to survive and persist in the human GI tract and exert an antiinflammatory effect [32], and a previous study showed the ability of other strains of *S. clausii* to increase the levels of the antiinflammatory cytokine IL‐10 and decrease the levels of the proinflammatory cytokines TNF‐α and IL‐6 [10]. The most interesting result is that probiotic administration was also able to significantly increase the IL‐10 content in portal plasma, compared to both C and F rats, thus showing a diet‐independent antiinflammatory effect, which was also evident in liver and skeletal muscle, with a significant decrease in tissue TNF‐α compared to F and C rats.

The deleterious effect of fructose on the liver, the primary site of fructose metabolism, and on the skeletal muscle cannot be attributed solely to the induction of the inflammatory state. It is known that increased fructose intake results in increased hepatic and muscular TAGs because of the activation of de novo lipogenesis pathway in the liver [33, 34, 35]. In agreement, we found an increased activity of the FAS enzyme, a de novo lipogenesis key enzyme [36], higher TAGs levels in both liver and skeletal muscle, and a higher degree of hepatic steatosis. Probiotic‐treated rats exhibited normal content of TAGs and no sign of hepatic steatosis.

In addition, fructose intake deeply alters the whole‐body glucose homeostasis, impairing the insulin sensitivity at the level of the liver and skeletal muscle [37]. The onset of insulin resistance is strictly correlated with inflammation and with the ectopic deposition of TAGs at the level of these two tissues. Indeed, the chronic low‐grade inflammation due to activation of the innate immune system and release of cytokines is associated with a whole‐body reduction in insulin sensitivity [38] and the ectopic TAGs deposition leads to the production of ceramide, which is known to shut down insulin signaling. In agreement, F rats exhibited systemic insulin resistance, while we here show for the first time that the administration of *S. clausii* SF174 spores was effective in preserving insulin sensitivity. We here propose that the mechanism underlying this effect is not only due to the reported ability of probiotics in reducing endotoxemia [39, 40, 41] but also to the specific antiinflammatory effect of *S. clausii* SF174 spores mediated by increased portal levels of IL‐10. In fact, it has been reported that IL‐10 prevents diet‐induced insulin resistance both in liver and skeletal muscle, through a reduction in the production of tissue IL‐6 and TNF‐α [42, 43, 44].

Mitochondria are the main cellular sites devoted to ATP production and lipid oxidation; therefore, the mitochondrial dysfunction could be an important determinant of the cellular fate of circulating lipids [45]. It is interesting to notice that in our experimental paradigm, fructose affects mitochondrial functioning differently in the liver and in the skeletal muscle. In the liver, F rats showed an increase in mitochondrial respiration with Complex I‐ and II‐linked substrates, but not only with Complex I‐linked substrates. The respiration was also increased in the presence of FCCP, an artificial uncoupler that dissociates the oxidation of substrates from the synthesis of ATP, thus suggesting that ATP synthase is not involved in the regulation of mitochondrial respiration, and in the presence of rotenone, an inhibitor of Complex I, suggesting that the point of regulation is located in the respiratory chain from Complex II onwards. This result is also confirmed by the increased activity of the Complex II recorded in F rats. On the contrary, in the skeletal muscle, fructose‐rich diet induced a decrease in mitochondrial maximal oxidative capacity with FAD‐ and NAD‐linked substrates, and in the presence of FCCP and rotenone. These results obtained after 6 weeks of high fructose diet are in agreement with our previous results, showing these different mitochondrial alterations in the liver and skeletal muscle even after a short‐term fructose diet [7, 25]. The different impact of fructose on the two organs can be explained considering that the liver is the major organ for fructose metabolism [46], receiving 80–90% of the fructose load coming from the diet [47], where fructose metabolism does not have any regulatory point, leading to a fast depletion of cellular ATP [48] with a following increase in metabolic signaling molecule AMP, that is known to stimulate mitochondrial activity [49]. This strongly impacts on the handling of energy in the liver, thus eliciting a compensatory response by the mitochondria, that increase their activity to counteract the ATP depletion. On the other hand, at the level of skeletal muscle mitochondria, the increased nutrient availability and the subsequent mitochondrial fuel overload triggers a counterregulatory mechanism that leads to the inhibition of the electron transport chain, as previously shown in an in vitro model of endothelial cells [50] and in L6 myotubes [51]. Therefore, we can speculate that the mechanisms behind the differential effects on mitochondrial activity in liver and skeletal muscle are different, depending on the type of substrate reaching the two organs, with fructose impacting on the liver and fatty acids impacting on skeletal muscle.

In this context, in which the fructose‐rich diet seems to have an opposite impact on liver and skeletal muscle mitochondrial energetics, it is interesting to notice that an increase in the oxidative damage to lipids, as shown by high TBARS levels, was found in both tissues. This oxidative imbalance is associated with the decrease in liver and skeletal muscle antioxidant defenses, indicated by the lower activity of enzymes such as catalase, SOD1 and SOD2. Usually, the increased oxidative damage in presence of mitochondrial alteration is also associated with higher production of ROS, considering that the mitochondria are the main site of ROS production [52], but this might not be the case. Indeed, the decreased mitochondrial respiratory capacity found in the skeletal muscle might induce an increased ROS production, considering that the Complex II is involved in redox control, as it is responsible for shuttling electrons from succinate to ubiquinone; thus, impairment in Complex II will hamper electron flow to Complex III and the quinone pool, thereby promoting oxidative stress, because electrons constantly leak from the respiratory chain to generate deleterious ROS [53]. On the other hand, in the presence of metabolic alterations, the liver is a major organ attacked by ROS [54] and the enzymatic antioxidant system is essential for cellular response to deal with oxidative stress under these conditions [55]. Therefore, the decrease in catalase, SOD1 and SOD2 activity could be the main cause of the oxidative stress exhibited by the liver of fructose fed rats, even in presence of the compensatory response that enhances mitochondrial respiration and Complex II activity.

In summary, our present results demonstrate for the first time the efficiency of *S. clausii* SF174 spores in improving the metabolic alterations induced in the liver and skeletal muscle by the fructose‐rich diet. Indeed, this probiotic prevents the onset of low‐grade inflammation and insulin resistance induced by fructose and preserves the mitochondria from changes in the respiratory capacity in both liver and skeletal muscle, thus maintaining unaltered the oxidative balance. One limitation of the study is the extrapolation of our present data obtained on the animal model to the human population. Therefore, testing *S. clausii* SF174 efficacy in preventing the unhealthy consequences of unbalanced diets in human trials is necessary before suggesting the formulation of new preparations for improving liver and skeletal muscle function.

## Ethics Statement

All the animal experiments were authorized by the Italian Health Ministry (137/2022‐PR) and approved by “Comitato Etico‐Scientifico per la Sperimentazione Animale” of the University of Naples “Federico II”. The procedures used in this work observe the animal ethics principles and regulations of the Italian Health Ministry.

## Conflicts of Interest

Ezio Ricca acts as a consultant for Gruppo Savio (Italy) that has the rights for the commercialization of strain SF174. Gruppo Savio (Italy) had no role in the design of the study, in the collection, analyses or interpretation of data, or in the writing of the manuscript. The other authors declare no conflicts of interest.

### Peer Review

The peer review history for this article is available at https://publons.com/publon/10.1002/mnfr.70063

## Supporting information



Supporting information

## Data Availability

All relevant data can be found within the article.

## References

[1] M. G. Saklayen , “The Global Epidemic of the Metabolic Syndrome,” Current Hypertension Reports 20, no. 2 (2018): 12, 10.1007/s11906-018-0812-z. 29480368 PMC5866840

[2] A. Nier , A. Brandt , D. Rajcic , et al., “Short‐Term Isocaloric Intake of a Fructose‐ but not Glucose‐Rich Diet Affects Bacterial Endotoxin Concentrations and Markers of Metabolic Health in Normal Weight Healthy Subjects,” Molecular Nutrition & Food Research 63, no. 6 (2019): 1800868, 10.1002/mnfr.201800868. 30570214 PMC6590154

[3] C. Guney , N. B. Bal , and F. Akar , “The Impact of Dietary Fructose on Gut Permeability, Microbiota, Abdominal Adiposity, Insulin Signaling and Reproductive Function,” Heliyon 9, no. 8 (2023): 18896, 10.1016/j.heliyon.2023.e18896. PMC10447940 37636431

[4] S. Dogan , M. Celikbilek , and K. Guven , “High Fructose Consumption Can Induce Endotoxemia,” Gastroenterology 143, no. 3 (2012): 29, 10.1053/j.gastro.2012.07.012. 22841729

[5] S. Jung , H. Bae , W. S. Song , and C. Jang , “Dietary Fructose and Fructose‐Induced Pathologies,” Annual Review of Nutrition 42 (2022): 45–66, 10.1146/annurev-nutr-062220-025831. PMC9904196 35995049

[6] R. Crescenzo , L. Cigliano , A. Mazzoli , et al., “Early Effects of a Low Fat, Fructose‐Rich Diet on Liver Metabolism, Insulin Signaling, and Oxidative Stress in Young and Adult Rats,” Frontiers in Physiology 9 (2018): 411, 10.3389/fphys.2018.00411. 29755364 PMC5932594

[7] C. Gatto , A. Di Porzio , R. Crescenzo , V. Barrella , S. Iossa , and A. Mazzoli , “Age‐Dependent Skeletal Muscle Mitochondrial Response to Short‐Term Increased Dietary Fructose,” Antioxidants (Basel) 12, no. 2 (2023): 299, 10.3390/antiox12020299. 36829857 PMC9951991

[8] S. Puebla‐Barragan and G. Reid , “Thirty‐Year Evolution of Probiotic Therapy,” Microbial Cell 6, no. 4 (2019): 184–196, 10.15698/mic2019.04.673. 30956971 PMC6444557

[9] L. R. Lopetuso , F. Scaldaferri , F. Franceschi , and A. Gasbarrini , “ *Bacillus clausii* and Gut Homeostasis: State of the Art and Future Perspectives,” Expert Review of Gastroenterology & Hepatology 10, no. 8 (2016): 943–948, 10.1080/17474124.2016.1200465. 27291780

[10] E. Ghelardi , Y. Abreu , A. T. Abreu , et al., “Current Progress and Future Perspectives on the Use of Bacillus clausii,” Microorganisms 10, no. 6 (2022): 1246, 10.3390/microorganisms10061246. 35744764 PMC9230978

[11] D. Mazzantini , M. Calvigioni , F. Celandroni , et al., “In Vitro Assessment of Probiotic Attributes for Strains Contained in Commercial Formulations,” Scientific Reports 12 (2022): 21640, 10.1038/s41598-022-25688-z. 36517529 PMC9751119

[12] H. T. Dang , D. M. Tran , T. T. B. Phung , et al., “Promising Clinical and Immunological Efficacy of *Bacillus clausii* Spore Probiotics for Supportive Treatment of Persistent Diarrhea in Children,” Scientific Reports 14, no. 1 (2024): 6422, 10.1038/s41598-024-56627-9. 38494525 PMC10944834

[13] R. Vázquez‐Frias , A. Consuelo‐Sánchez , C. P. Acosta‐Rodríguez‐Bueno , et al., “Efficacy and Safety of the Adjuvant Use of Probiotic *Bacillus clausii* Strains in Pediatric Irritable Bowel Syndrome: A Randomized, Double‐Blind, Placebo‐Controlled Study,” Paediatric Drugs 25, no. 1 (2023): 115–126, 10.1007/s40272-022-00536-9. 36380186 PMC9666949

[14] K. Rea , J. Colom , E. A. Simon , et al., “Evaluation of *Bacillus clausii* CSI08, *Bacillus megaterium* MIT411 and a *Bacillus* Cocktail on Gastrointestinal Health: A Randomised, Double‐Blind, Placebo‐Controlled Pilot Study,” Beneficial Microbes 14, no. 2 (2023): 165–182, 10.3920/BM2022.0117. 37026366

[15] E. Khokhlova , J. Colom , A. Simon , et al., “Immunomodulatory and Antioxidant Properties of a Novel Potential Probiotic *Bacillus clausii* CSI08,” Microorganisms 11 (2023): 240, 10.3390/microorganisms11020240. 36838205 PMC9962608

[16] A. Saggese , V. Barrella , A. Di Porzio , et al., “Protective Role of Cells and Spores of *Shouchella clausii* sf174 Against Fructose‐Induced Gut Dysfunctions in Small and Large Intestine,” Journal of Nutritional Biochemistry 133 (2024): 109706, 10.1016/j.jnutbio.2024.109706. 39053859

[17] Y. Ruan , P. Yuan , P. Li , et al., “Tingli Dazao Xiefei Decoction Ameliorates Asthma in Vivo and In Vitro From Lung to Intestine by Modifying NO–CO Metabolic Disorder Mediated Inflammation, Immune Imbalance, Cellular Barrier Damage, Oxidative Stress and Intestinal Bacterial Disorders,” Journal of Ethnopharmacology 313 (2023): 116503, 10.1016/j.jep.2023.116503. 37116727

[18] L. Huang , Y. Li , R. Tang , et al., “Bile Acids Metabolism in the Gut‐Liver Axis Mediates Liver Injury During Lactation,” Life Sciences 338 (2024): 122380, 10.1016/j.lfs.2023.122380. 38142738

[19] J. Li , Y. Chen , S. Zhang , et al., “Purslane (*Portulaca oleracea* L.) Polysaccharide Attenuates Carbon Tetrachloride‐Induced Acute Liver Injury by Modulating the Gut Microbiota in Mice,” Genomics 117, no. 1 (2025): 110983, 10.1016/j.ygeno.2024.110983. 39734003

[20] W. Li , J. Wu , D. Xiang , et al., “Micelles Loaded With Puerarin and Modified With Triphenylphosphonium Cation Possess Mitochondrial Targeting and Demonstrate Enhanced Protective Effect Against Isoprenaline‐Induced H9c2 Cells Apoptosis,” International Journal of Nanomedicine 14 (2019): 8345–8360, 10.2147/IJN.S219670. 31695371 PMC6814317

[21] J. He , X. Feng , Y. Liu , et al., “Graveoline Attenuates D‐GalN/LPS‐Induced Acute Liver Injury via Inhibition of JAK1/STAT3 Signaling Pathway,” Biomedicine & Pharmacotherapy 177 (2024): 117163, 10.1016/j.biopha.2024.117163. 39018876

[22] A. R. Maia , R. Reyes‐Ramírez , and M. Pizarro‐Guajardo , “Nasal Immunization With the c‐Terminal Domain of bcla3 Induced Specific Igg Production and Attenuated Disease Symptoms in Mice Infected With C*lostridioides difficile* Spores,” International Journal of Molecular Sciences 21, no. 18 (2020): 6696, 10.3390/ijms21186696. 32933117 PMC7555657

[23] A. Saggese , R. Isticato , G. Cangiano , et al., “CotG‐Like Modular Proteins Are Common Among Spore‐Forming Bacilli,” Journal of Bacteriology 198, no. 10 (2016): 1513–1520, 10.1128/jb.00023-16. 26953338 PMC4859607

[24] M. A. Abdul‐Ghani , M. Matsuda , B. Balas , and R. A. DeFronzo , “Muscle and Liver Insulin Resistance Indexes Derived from the Oral Glucose Tolerance Test,” Diabetes Care 30, no. 1 (2007): 89–94, 10.2337/dc06-1519. 17192339

[25] A. Mazzoli , C. Gatto , R. Crescenzo , et al., “Prolonged Changes in Hepatic Mitochondrial Activity and Insulin Sensitivity by High Fructose Intake in Adolescent Rats,” Nutrients 13, no. 4 (2021): 1370, 10.3390/nu13041370. 33921866 PMC8073121

[26] M. Spinazzi , A. Casarin , V. Pertegato , L. Salviati , and C. Angelini , “Assessment of Mitochondrial Respiratory Chain Enzymatic Activities on Tissues and Cultured Cells,” Nature Protocols 7, no. 6 (2012): 1235–1246, 10.1038/nprot.2012.058. 22653162

[27] A. Mazzoli , G. Donadio , M. Lanzilli , et al., “ *Bacillus megaterium* SF185 Spores Exert Protective Effects against Oxidative Stress In Vivo and In Vitro,” Scientific Reports 9, no. 1 (2019): 12082, 10.1038/s41598-019-48531-4. 31427655 PMC6700169

[28] L. Flohé and F. F. Otting , “Superoxide Dismutase Assays,” Methods in Enzymology 105 (1984): 93–104, 10.1016/s0076-6879(84)05013-8. 6328209

[29] R. Crescenzo , M. S. Spagnuolo , R. Cancelliere , et al., “Effect of Initial Aging and High‐Fat/High‐Fructose Diet on Mitochondrial Bioenergetics and Oxidative Status in Rat Brain,” Molecular Neurobiology 56, no. 11 (2019): 7651–7663, 10.1007/s12035-019-1617-z. 31089964

[30] J. Todoric , G. Di Caro , S. Reibe , et al., “Fructose Stimulated De Novo Lipogenesis Is Promoted by Inflammation,” Nature Metabolism 2, no. 10 (2020): 1034–1045, 10.1038/s42255-020-0261-2. PMC8018782 32839596

[31] I. San‐Millán , “The Key Role of Mitochondrial Function in Health and Disease,” Antioxidants (Basel) 12, no. 4 (2023): 782, 10.3390/antiox12040782. 37107158 PMC10135185

[32] E. Ghelardi , F. Celandroni , S. Salvetti , et al., “Survival and Persistence of *Bacillus clausii* in the Human Gastrointestinal Tract Following Oral Administration as Spore‐Based Probiotic Formulation,” Journal of Applied Microbiology 119, no. 2 (2015): 552–559, 10.1111/jam.12848. 25973914

[33] R. Crescenzo , F. Bianco , P. Coppola , et al., “Increased Skeletal Muscle Mitochondrial Efficiency in Rats With Fructose‐Induced Alteration in Glucose Tolerance,” British Journal of Nutrition 110, no. 11 (2013): 1996–2003, 10.1017/s0007114513001566. 23693085

[34] S. Softic , K. L. Stanhope , J. Boucher , et al., “Fructose and Hepatic Insulin Resistance,” Critical Reviews in Clinical Laboratory Sciences 57, no. 5 (2020): 308–322, 10.1080/10408363.2019.1711360. 31935149 PMC7774304

[35] A. Mazzoli , A. Di Porzio , C. Gatto , et al., “Skeletal Muscle Insulin Resistance and Adipose Tissue Hypertrophy Persist Beyond the Reshaping of Gut Microbiota in Young Rats Fed a Fructose‐Rich Diet,” Journal of Nutritional Biochemistry 113 (2023): 109247, 10.1016/j.jnutbio.2022.109247. 36496062

[36] T. Mashima , H. Seimiya , and T. Tsuruo , “De Novo Fatty‐Acid Synthesis and Related Pathways as Molecular Targets for Cancer Therapy,” British Journal of Cancer 100, no. 9 (2009): 1369–1372, 10.1038/sj.bjc.6605007. 19352381 PMC2694429

[37] S. Softic , D. E. Cohen , and C. R. Kahn , “Role of Dietary Fructose and Hepatic De Novo Lipogenesis in Fatty Liver Disease,” Digestive Diseases and Sciences 61, no. 5 (2016): 1282–1293, 10.1007/s10620-016-4054-0. 26856717 PMC4838515

[38] M. Krssak and M. Roden , “The Role of Lipid Accumulation in Liver and Muscle for Insulin Resistance and Type 2 Diabetes Mellitus in Humans,” Reviews in Endocrine & Metabolic Disorders 5, no. 2 (2004): 127–134, 10.1023/b:remd.0000021434.98627.dc. 15041788

[39] M. Le Barz , F. F. Anhê , T. V. Varin , et al., “Probiotics as Complementary Treatment for Metabolic Disorders,” Diabetes & Metabolism Journal 39, no. 4 (2015): 291–303, 10.4093/dmj.2015.39.4.291. 26301190 PMC4543192

[40] C. S. Hampe and C. L. Roth , “Probiotic Strains and Mechanistic Insights for the Treatment of Type 2 Diabetes,” Endocrine 58, no. 2 (2017): 207–227, 10.1007/s12020-017-1433-z. 29052181

[41] S. Firouzi , H. A. Majid , A. Ismail , N. A. Kamaruddin , and M. Y. Barakatun‐Nisak , “Effect of Multi‐Strain Probiotics (Multi‐Strain Microbial Cell Preparation) on Glycemic Control and Other Diabetes‐Related Outcomes in People with Type 2 Diabetes: A Randomized Controlled Trial,” European Journal of Nutrition 56, no. 4 (2017): 1535–1550, 10.1007/s00394-016-1199-8. 26988693

[42] D. E. Cintra , J. R. Pauli , E. P. Arau ´jo , et al., “Interleukin‐10 Is a Protective Factor Against Diet‐Induced Insulin Resistance in Liver,” Journal of Hepatology 48 (2008): 628–637, 10.1016/j.jhep.2007.12.017. 18267346

[43] E. G. Hong , H. J. Ko , Y. R. Cho , et al., “Interleukin‐10 Prevents Diet‐Induced Insulin Resistance by Attenuating Macrophage and Cytokine Response in Skeletal Muscle,” Diabetes 58, no. 11 (2009): 2525–2535, 10.2337/db08-1261. 19690064 PMC2768157

[44] S. Dagdeviren , D. Y. Jung , R. H. Friedline , et al., “IL‐10 Prevents Aging‐Associated Inflammation and Insulin Resistance in Skeletal Muscle,” The FASEB Journal 31, no. 2 (2017): 701–710, 10.1096/fj.201600832R. 27811060 PMC5240661

[45] R. Crescenzo , F. Bianco , A. Mazzoli , et al., “A Possible Link Between Hepatic Mitochondrial Dysfunction and Diet‐Induced Insulin Resistance,” European Journal of Nutrition 56, no. 4 (2017): 1535–1550, 10.1007/s00394-015-1073-0. 26476631

[46] S. A. Hannou , D. E. Haslam , N. M. McKeown , and M. A. Herman , “Fructose Metabolism and Metabolic Disease,” Journal of Clinical Investigation 128, no. 2 (2018): 545–555, 10.1172/jci96702. 29388924 PMC5785258

[47] K. L. Stanhope , “Sugar Consumption, Metabolic Disease and Obesity: The State of the Controversy,” Critical Reviews in Clinical Laboratory Sciences 53, no. 1 (2016): 52–67, 10.3109/10408363.2015.1084990. 26376619 PMC4822166

[48] Z. Khitan and D. H. Kim , “Fructose: A Key Factor in the Development of Metabolic Syndrome and Hypertension,” Journal of Nutrition and Metabolism 2013 (2013): 682673, 10.1155/2013/682673. 23762544 PMC3677638

[49] P. Prasun , I. Ginevic , and K. Oishi , “Mitochondrial Dysfunction in Nonalcoholic Fatty Liver Disease and Alcohol Related Liver Disease,” Translational Gastroenterology and Hepatology 6 (2021): 4, 10.21037/tgh-20-125. 33437892 PMC7792990

[50] C. Hansen , K. Olsen , H. Pilegaard , et al., “High Metabolic Substrate Load Induces Mitochondrial Dysfunction in Rat Skeletal Muscle Microvascular Endothelial Cells,” Physiological Reports 9, no. 14 (2021): 14855, 10.14814/phy2.14855. PMC8290479 34288561

[51] N. Jaiswal , C. K. Maurya , D. Arha , et al., “Fructose Induces Mitochondrial Dysfunction and Triggers Apoptosis in Skeletal Muscle Cells by Provoking Oxidative Stress,” Apoptosis 20, no. 7 (2015): 930–947, 10.1007/s10495-015-1128-y. 25913123

[52] P. Hernansanz‐Agustín and J. A. Enríquez , “Generation of Reactive Oxygen Species by Mitochondria,” Antioxidants 10, no. 3 (2021): 415, 10.3390/antiox10030415. 33803273 PMC8001687

[53] Z. Zhou , A. Ma , T. M. Moore , et al., “Drp1 Controls Complex II Assembly and Skeletal Muscle Metabolism by Sdhaf2 Action on Mitochondria,” Science Advances 10, no. 14 (2024): adl0389, 10.1126/sciadv.adl0389. PMC10990287 38569044

[54] V. Sánchez‐Valle , N. C. Chávez‐Tapia , M. Uribe , and N. Méndez‐Sánchez , “Role of Oxidative Stress and Molecular Changes in Liver Fibrosis: A Review,” Current Medicinal Chemistry 19, no. 28 (2012): 4850–4860, 10.2174/092986712803341520. 22709007

[55] J. Medina and R. Moreno‐Otero , “Pathophysiological Basis for Antioxidant Therapy in Chronic Liver Disease,” Drugs 65, no. 17 (2005): 2445–2461, 10.2165/00003495-200565170-00003. 16296871

