# Supplementary material for: Fructose‐Induced Impairment of Liver and Skeletal Muscle Metabolism Is Prevented by Administration of Shouchella clausii Spores by Preserving Mitochondrial Function and Insulin Sensitivity
Source: Mol Nutr Food Res. 2025 Apr 10;69(11):e70063. doi: 10.1002/mnfr.70063 (PMC12128003; doi:10.1002/mnfr.70063)
Supplement: Supplementary file 1 — Supporting information [file MNFR-69-e70063-s001.docx]

**Supplementary materials**

**Table S1.** Composition of experimental diets.

| Component (g/100g) Control diet Fructose diet |
| --- |

Standard Chow^a^ 50.5 50.5

Sunflower Oil 1.5 1.5

Casein 9.2 9.2

Alphacel 9.8 9.8

Starch 20.4 ---

Fructose --- 20.4

Water 6.4 6.4

AIN-76 mineral mix 1.6 1.6

AIN-76 vitamin mix 0.4 0.4

Choline 0.1 0.1

Methionine 0.1 0.1

Gross Energy Density (kJ/g) 17.2 17.2

Metabolisable Energy Density (kJ/g) ^b^ 11.1 11.1

Proteins (% Metabolisable energy) 29.0 29.0

Lipids (% Metabolisable energy) 10.6 10.6

Carbohydrates (% Metabolisable energy) 60.4 60.4

Of which:

Fructose --- 30.0

Starch 52.8 22.8

Sugars 7.6 7.6

| ^a^Mucedola 4RF21; Italy ^b^Estimated by computation using values (kJ/g) for energy content as follows: proteins 16.736, lipids 37.656 e carbohydrates 16.736 |
| --- |

**Fig. S1** Cumulative food intake (A) and body weight (B) in rats fed control diet (C), fructose-rich diet (F), and fructose-rich diet plus spores of *S. clausii* SF174 (SF174) for 6 weeks. Values are the means ± SEM of eight different rats.
